# Lightweight Brain Tumor Segmentation Through Wavelet-Guided Iterative Axial Factorization Attention

**DOI:** 10.3390/brainsci15060613

**Published:** 2025-06-06

**Authors:** Yueyang Zhong, Shuyi Wang, Yuqing Miao, Tao Zhang, Haoliang Li

**Affiliations:** 1 School of Health Science and Engineering, University of Shanghai for Science and Technology, Shanghai 200093, China; 233352436@st.usst.edu.cn (Y.Z.); 233352437@st.usst.edu.cn (T.Z.); 232322256@st.usst.edu.cn (H.L.); 2 School of Materials and Chemistry, University of Shanghai for Science and Technology, Shanghai 200093, China; yqmiao@usst.edu.cn

**Keywords:** brain tumor segmentation, discrete wavelet transform, axial transformer, computational efficiency

## Abstract

Background/Objectives: The accurate and efficient segmentation of brain tumors from 3D MRI data remains a significant challenge in medical imaging. Conventional deep learning methods, such as convolutional neural networks and transformer-based models, frequently introduce significant computational overhead or fail to effectively represent multi-scale features. Methods: This paper presents a lightweight deep learning framework that uses adaptive discrete wavelet decomposition and iterative axial attention to improve 3D brain tumor segmentation. The wavelet decomposition module effectively captures multi-scale information by breaking it down into frequency sub-bands, thereby the mitigating detail loss often associated with standard downsampling methods. Ablation studies confirm that this module enhances segmentation accuracy, particularly in preserving the finer structural details of tumor components. Simultaneously, the iterative axial factorization attention reduces the computational burden of 3D spatial modeling by processing attention sequentially along individual axes, preserving long-range interdependence while consuming minimal resources. Results: Our model performs well on the BraTS2020 and FeTS2022 datasets with average Dice scores of 85.0% and 88.1%, with our competitive results using only 5.23 million parameters and 9.75 GFLOPs. In comparison to state-of-the-art methods, it effectively balances accuracy and efficiency, making it suitable for resource-constrained clinical applications. Conclusions: This study underscores the advantages of integrating frequency-domain analysis with optimized attention mechanisms, paving the way for scalable, high-performance medical image segmentation algorithms with broader clinical diagnostic applications.

## 1. Introduction

As the most common primary malignant tumors in the central nervous system, gliomas are highly heterogeneous, aggressive, and have a poor clinical prognosis. Accurate imaging assessment is crucial for surgical planning and efficacy monitoring [1]. Multimodal magnetic resonance imaging (MRI) provides multidimensional information on tumor anatomy and function through T1-weighted, T1-enhanced, T2-weighted, and FLAIR sequences, but manually outlining the tumor region is time consuming and susceptible to subjective factors [2]. In recent years, deep learning techniques have significantly promoted the automation of medical image segmentation; however, 3D glioma segmentation still faces many challenges: variable tumor morphology, fuzzy boundaries, large-scale variations, and complex semantic features in different sub-regions such as enhanced tumors and peri-tumor edemas [3]. How to balance the computational efficiency and segmentation accuracy of the model while effectively modeling long-range dependencies and multi-scale features has become a core problem to be solved in this field.

Traditional convolutional neural networks (CNNs) dominate medical image segmentation by virtue of their local feature extraction capability. The encoder-decoder architecture represented by U-Net [4] fuses shallow details with deep semantic information through jump connections and performs well in the brain tumor segmentation task. Subsequent studies have further optimized the performance by introducing designs such as residual connectivity [5], attention mechanisms [6], and adaptive normalization [7]. However, traditional CNNs are limited by local receptive fields, which makes it difficult to capture the global spatial correlation of tumors, and are especially ineffective in segmenting irregular morphology or small-sized lesions [8]. In addition, the high-resolution nature of 3D medical images leads to a surge in the number of model parameters and computational complexity, which also limits the efficiency of their deployment in clinical environments [9].

In order to break through the limitations of CNN, researchers have attempted to introduce Transformer [10] into the field of medical image segmentation. Vision Transformer (ViT) [11] models the global context through a self-attentive mechanism and demonstrates excellent performance in natural image tasks. TransUNet [8] embedded ViT into the bottleneck layer of U-Net for the first time, which utilizes Transformer to encode long-range dependencies while preserving the local feature extraction capability of CNN. However, the high computational complexity and data dependency of ViT limit its application in 3D medical imaging: the original ViT needs to divide the image into fixed-size patches, which leads to the loss of spatial information, and the pre-training weights depend on large-scale datasets [12]. Swin UNETR [13] reduces computational complexity through the mechanism of layered-windowed self-attention and demonstrates excellent performance, but its parameter count is still high, which makes it difficult to meet the lightweight demand. Recent studies have proposed hybrid architectures, such as TransBTS [14], which combines 3D CNN and Transformer in the encoder, which improves the segmentation accuracy; however, the efficiency of multi-stage feature fusion is insufficient, and the decoupling of multi-scale frequency information is not fully considered.

Existing methods still have significant limitations in the following aspects: the traditional downsampling operation represented by pooling or stepwise convolution is prone to lead to the loss of high-frequency details, while frequency-domain analysis methods, such as wavelet transform, can preserve multiscale features, but their nondiminutability restricts the ability of end-to-end optimization [15]; the computational complexity of the 3D global self-attention grows squarely with the number of voxels, which makes it difficult to be directly applied to high-resolution MRI data [16]; most existing decoders rely on simple upsampling and jump connections and lack dynamic calibration and structured fusion of multi-scale features, leading to blurring of boundary segmentation and false-positive problems [17]. In addition, most models do not fully consider the inter-class imbalance problem, especially in small target regions such as enhanced tumors (ETs), which are prone to under-segmentation [18].

Furthermore, the deployment of deep learning models in clinical settings, such as intraoperative surgical navigation systems or resource-constrained diagnostic centers, imposes stringent requirements on computational efficiency [19]. Surgical navigation, for instance, demands near real-time segmentation feedback to guide interventions, necessitating models with low latency and minimal computational footprint.

To address the above motivation, the study in this paper proposes a lightweight 3D medical image segmentation framework for gliomas, which we name the Wavelet-guided Iterative Axial Factorization (WIAF) model. The main design includes an adaptive discrete wavelet domain feature decoupling module (AWD-FD), which separates multi-frequency-band features via a three-dimensional discrete wavelet transform and incorporates attentional gating to dynamically augment the task-relevant frequency components. Our proposed iterative axial decomposition of attention mechanism (IAFT) significantly reduces the computational time complexity by decomposing the 3D self-attention into serialized local computations in the height, width, and depth directions; the model employs a multiscale feature fusion decoder (MSFFD) to enhance the boundary segmentation accuracy through axial attention-guided hierarchical feature alignment with a progressive upsampling strategy. Our experiments show that the model achieves a state-of-the-art performance level on the BraTS2020 and FeTS2022 datasets, respectively, while the number of covariates is only 5.23 M and the computation amount is 9.75 GFLOPs, which is a significant advantage.

The contributions of this paper can be summarized as follows:

- Combining 3D wavelet transform with the wavelet coefficient gating mechanism to realize end-to-end multi-band feature decoupling and dynamic calibration.
- Proposal of an iterative attention mechanism for axial decomposition to efficiently model long-range dependencies while preserving local geometric structures.
- Design of a lightweight multi-scale decoder to balance the computational efficiency and segmentation accuracy through a joint channel-space optimization strategy.

The remainder of this paper is structured as follows: Section 2 reviews related research methods and progress; Section 3 details the model architecture and key technologies; Section 4 demonstrates the experimental design and result analysis; Section 5 discusses the model limitations and future directions. The source code of the proposed model is available in the Supplementary Materials.

## 2. Related Work

### 2.1. Convolutional Neural Network-Based Medical Image Segmentation Methods

Since the proposal of U-Net [4], the encoder-decoder architecture has become the dominant paradigm for medical image segmentation. For the task of 3D glioma segmentation, 3D U-Net [20] extends the modeling capability of traditional U-Net by introducing 3D convolution, but its restricted receptive field leads to insufficient accuracy for small-size tumor segmentation. Subsequent studies, such as Res-UNet [21] optimized gradient propagation by residual connection, and Dense-UNet [22] introduced the design of dense connection but failed to effectively solve the multi-scale feature fusion problem caused by tumor heterogeneity.

### 2.2. Attention Mechanism and Feature Calibration Strategy

The attention mechanism significantly improves the performance of medical image segmentation through dynamic feature weighting. The nnFormer proposed by Zhou et al. [23] utilizes a combination of interleaved convolution and self-attention operations and also introduces a self-attention mechanism based on local and global volumes to learn the volume representation. TransBTS proposed by Wang et al. [14] learned the volume representation through the Transformer bottleneck layer to model global dependencies, but multi-stage feature alignment is inefficient. Attention U-Net proposed by Oktay et al. [24] introduces gated attention into encoder-decoder jump connections. TransUNet [8] embeds Vision Transformer into the encoder to capture long-range dependencies using self-attention, but its two-dimensional patch division leads to loss of 3D spatial continuity. UNETR proposed by Hatamizadeh et al. [9] uses a pure Transformer architecture, which is prone to overfitting on small datasets. To balance the local details and global context, Zhang et al. [25] designed a hybrid CNN-Transformer architecture, which still faces high complexity in its multi-branch computation.

### 2.3. Application of Wavelet Transform in Medical Image Analysis

Wavelet transform shows unique advantages in medical image denoising and feature decoupling by virtue of its multi-resolution analysis capability. Liu et al. [26] proposed MWCNN to integrate discrete wavelet transform into a CNN downsampling layer, but the non-microscopic wavelet operation restricted the end-to-end optimization. Kang et al. [27] constructed a wavelet residual network to suppress the noise propagation by frequency domain decomposition; however, the high-frequency information is directly discarded and easily leads to the loss of edge details. Zhao et al. [28] proposed WRANet using learnable wavelet basis functions to achieve a dynamic frequency band selection. Huang et al. [29] further designed a reversible wavelet network, WINNet, to enhance the feature robustness through the joint frequency domain-space domain optimization.

### 2.4. Lightweight Transformer Architecture Design

To address the high computational complexity problem of traditional Transformer, the axial attention mechanism has been widely explored. Axial-DeepLab proposed by Wang et al. [30] decomposed the 2D self-attention into 1D computation in the height and width directions. Valanarasu et al. [31] proposed MedT, which introduced gated axial attention and dynamically adjusted the positional coding contribution through learnable gating parameters. Perera et al. [32] proposed SegFormer3D with a hierarchical Transformer with a full MLP decoder, with a parameter count of only 4.5 M, and achieved similar performance to nnFormer [23]. In addition, EfficientViT [33] reduced memory consumption by cascading group attention, but its channel compression strategy leads to high-frequency feature loss.

### 2.5. Multi-Scale Feature Fusion

Feature fusion and information interaction play a very important role in segmentation boundary accuracy. CE-Net, proposed by Gu et al. [34], introduces a feature pyramid module to enhance multiscale context awareness, but its cascaded upsampling strategy creates a memory bottleneck. CA-Net proposed by Gu et al. [35], in order to achieve more accurate and interpretable medical image segmentation, calibrates the encoder features by introducing a joint spatial channel, and the scaled APRNet proposed by Zhuang et al. [36] uses 3D anisotropic pyramid convolution reversible residual sequences to capture multimodal image inter-dimensional information. Poudel et al. [37] designed a multiscale attention mechanism to expand the receptive field by expanding the convolution, but it is not sufficiently adapted to irregular tumor morphology.

Compared with existing studies, the key difference of this study is that the AWD-FD module proposed in this paper fully considers the importance of decoupling the frequency domain features, and adaptively enhances the task-relevant frequency components through a dynamic gating mechanism. The proposed IAFT module solves the problem of insufficient adaptation of traditional CNNs to irregular tumor morphology through an axial decomposition strategy that efficiently captures long-range dependencies while preserving local geometric structures. The overall framework achieves accurate modeling of 3D voxel-level spatial correlations while reducing the number of parameters and computational effort through an axial attention-guided hierarchical feature alignment strategy.

## 3. Methodology

### 3.1. Overview of the Proposed Model

**Figure 1:**
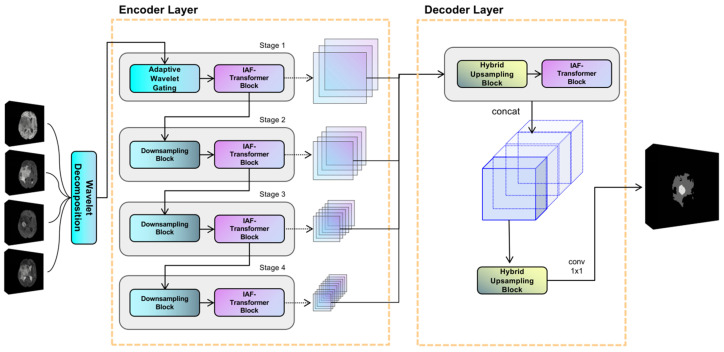
Overview of the proposed WIAF model architecture, highlighting the key components and their interactions for processing 3D medical imaging data.

The proposed model utilizes an encoder-decoder structure, and its overall framework is illustrated in Figure 1. The encoder is composed of four hierarchical stages for extracting multiscale features. Notably, the first stage uniquely incorporates an adaptive discrete wavelet domain feature decoupling (AWD-FD) design to process the initial input, while subsequent stages utilize standard convolutional downsampling. Each stage integrates an iterative axial factorization transformer (IAFT) module. The decoder is designed with multi-scale feature fusion, which aggregates features from different encoder stages and restores spatial resolution.

In the encoder, the initial stage implements the three-dimensional discrete wavelet transform to decompose the input volume into eight wavelet coefficients, ranging from low to high frequencies along the three orthogonal axes: height, width, and depth. Subsequent stages progressively reduce the feature sampling rate using segmented convolution while expanding the channel dimensions. It is evident that all stages employ IAFT blocks to model long-range dependencies through axial decomposition. The decoder upsamples the feature mappings to a uniform resolution and combines them along the channel dimensions through a tensor splicing operation. The convolutional layer then outputs the final prediction.

### 3.2. Adaptive Discrete Wavelet Domain Feature Decoupling

**Figure 2:**
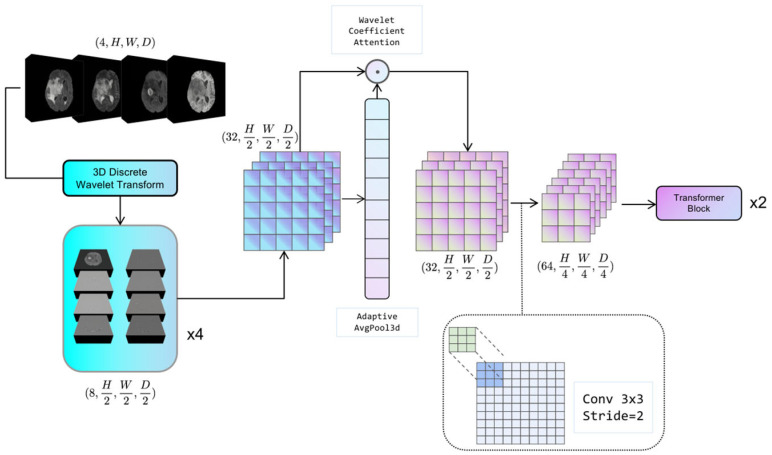
The schematic of Adaptive Wavelet Domain Feature Decoupling (AWD-FD) module. It depicts the process of feature decomposition and integration in the wavelet domain for multi-scale feature extraction.

The configuration of the adaptive discrete wavelet domain feature decoupling module is illustrated in Figure 2. The system under consideration comprises two distinct components: a non-learnable wavelet decomposition part and a learnable wavelet coefficient attention gating and downsampling part. The present study serializes the unlearnable part of the module along with the non-random transforms into the Hierarchical Data Format (HDF5) format [38], which is a file format for organizing and storing large amounts of data. This approach effectively mitigates the problem of non-micro-miniature wavelet transforms consuming model computation resources. The learnable part is trained as part of the backbone network.

#### 3.2.1. Discrete Wavelet Decomposition

For a given 3D medical image tensor $X\in {\mathbb{R}}^{C\times H\times W\times D}$, the Haar wavelet transform is utilized, where its corresponding low-pass filter *ϕ* and high-pass filter *ψ* are applied along each axis to decompose the tensor into eight wavelet coefficients, denoted as Equation (1):

(1) $$\begin{array}{l} X_{LLL}=\phi_{H}{}^{\circ}\phi_{W}{}^{\circ}\phi_{D}(X),\;X_{LLH}=\phi_{H}{}^{\circ}\phi_{W}{}^{\circ}\psi_{D}(X),\\ X_{LHL}=\phi_{H}{}^{\circ}\psi_{W}{}^{\circ}\phi_{D}(X),\;X_{LHH}=\phi_{H}{}^{\circ}\psi_{W}{}^{\circ}\psi_{D}(X),\\ X_{HLL}=\psi_{H}{}^{\circ}\phi_{W}{}^{\circ}\phi_{D}(X),\;X_{HLH}=\psi_{H}{}^{\circ}\phi_{W}{}^{\circ}\psi_{D}(X),\\ X_{HHL}=\psi_{H}{}^{\circ}\psi_{W}{}^{\circ}\phi_{D}(X),\;X_{HHH}=\psi_{H}{}^{\circ}\psi_{W}{}^{\circ}\psi_{D}(X), \end{array}$$

where ∘ denotes the convolution operation. For each mode of the image, it is decomposed into 8 wavelet coefficients; if the input 3D medical image contains C modes, C is the number of channels. The combined output of wavelet coefficients of all modes can be expressed as $X_{\mathrm{sub}}\in {\mathbb{R}}^{8C\times \frac{H}{2}\times \frac{W}{2}\times \frac{D}{2}}$.

#### 3.2.2. Adaptive Feature Recalibration

This section is used to dynamically adjust the sensitivity of the model to different wavelet coefficients by performing Global Average Pooling (GAP) on *Xsub* and generating learnable channel weight vectors $w\in {\mathbb{R}}^{8C}$ at the Multi-Layer Perceptron (MLP) layer. In Equation (2), W_1_ and W_2_ denote the two parameter matrices in the fully connected neural network:

(2) $$w=\sigma (W_{2}\cdot \mathrm{ReLU}(W_{1}\cdot \mathrm{GAP}(X_{\mathrm{sub}}))$$

The symbol “*σ*” denotes the sigmoid activation function, which is employed to generate weights ranging from 0 to 1. The recalibrated feature calculation formula is illustrated by Equation (3):

(3) $$\tilde{X}=X_{\mathrm{sub}}⊙w$$

In this context, ⊙ is used to denote the Hadamard product, defined as the element-by-element multiplication of the matrix. The module enables the model to adaptively suppress redundant information and enhance task-relevant frequency components.

### 3.3. Iterative Axial Factorization Transformer

In 3D medical image segmentation tasks, traditional global self-attention mechanisms face the challenge of high computational complexity. To solve this problem, this study proposes the IAFT module, which achieves efficient feature modeling through axial decomposition and iterative optimization. The core idea is to decompose the 3D spatial attention into serialized local attention computations in three orthogonal directions (height H, width W, and depth D), which is mainly used to balance computational efficiency and global context modeling.

**Figure 3:**
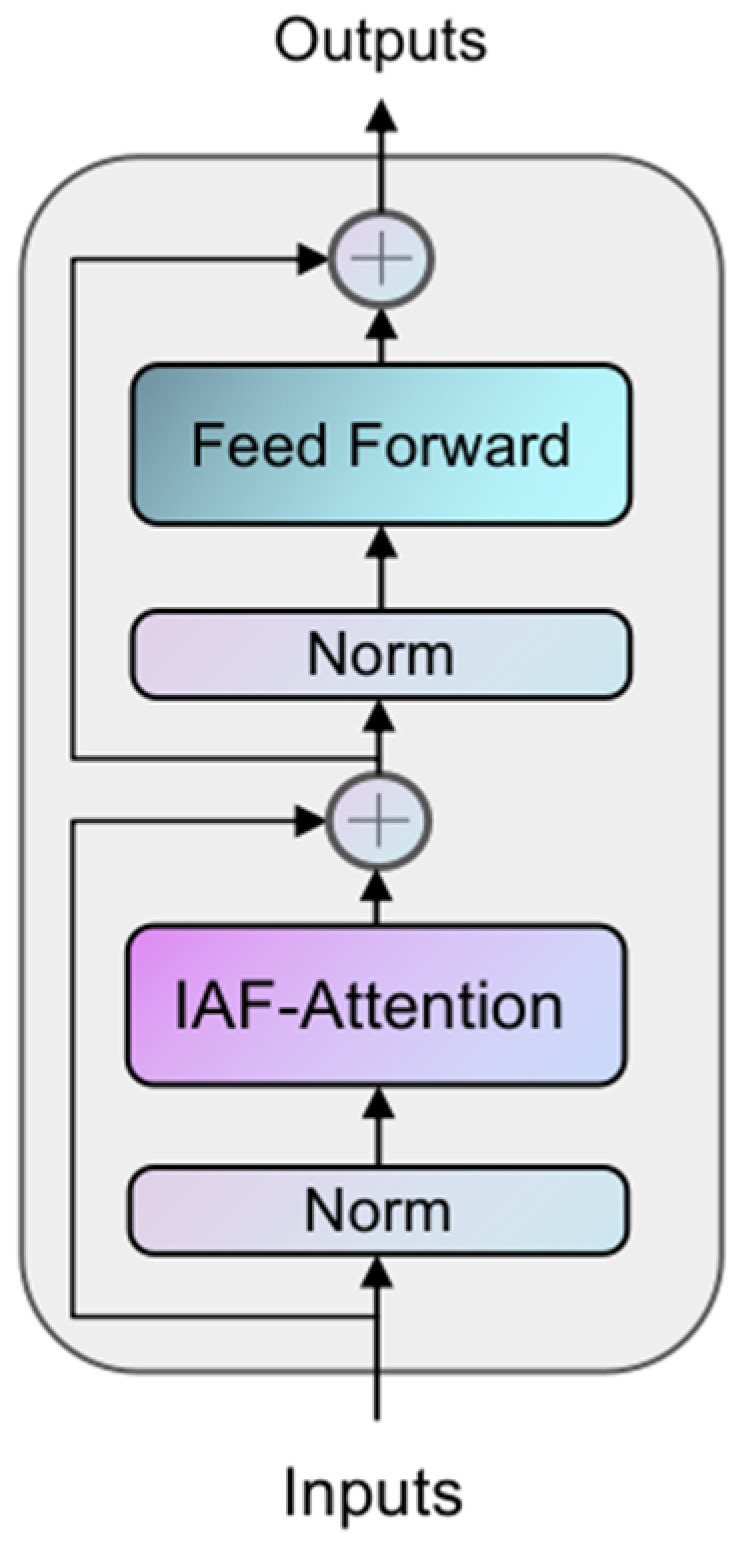
The architecture of the IAFT Module. Each block contains an Iterative Axial Factorization Attention (IAF-Attention), and MLP and incorporates a residual linkage design.

The structure of the IAFT module is shown in Figure 3, where the input tensor is layer-normalized and input into the Iterative Axial Factorization Attention; then, multi-layer level feature fusion is achieved by residual connection.

Given an input tensor X of shape (B, N, C), where N is defined as H × W × D, for the axis α, the dimensions are aligned through the operation of tensor reshaping to isolate the vector Z in the direction of α. To illustrate, the tensor is initially transformed along the height axis H into a tensor Zh of shape (W × D, H, C). Each tensor Z_α_ reshaped along the axis possesses distinct Query, Key, and Value components, which are derived through the learnable parameter matrices WQ, WK, and WV, respectively. The Qα, Kα, and Vα components in each axial direction are divided into heads according to the specified num_heads before performing the standard dot-product scaled attention computation as per the practice of multi-head self-attention, whose formulas are shown in (4):

(4) $${\mathrm{Attn}}_{\alpha }(Q_{\alpha },K_{\alpha },V_{\alpha })=\mathrm{Softmax}\left(\frac{Q_{\alpha }K_{\alpha }^{T}}{\sqrt{C}}\right)V_{\alpha }$$

where $\sqrt{c}$ is the scaling factor and C is equal to the feature dimension of the transformer block divided by num_heads. The outputs of the three axial iterations are sequentially summarized by residual concatenation, the representation of which is given in Equation (5):

(5) $$Z^{\left(t+1\right)}=Z^{\left(t\right)}+silu(\sum_{\alpha }{\mathrm{Attn}}_{\alpha }^{\left(t\right)}(Z_{\alpha }^{\left(t\right)}))$$

**Figure 4:**
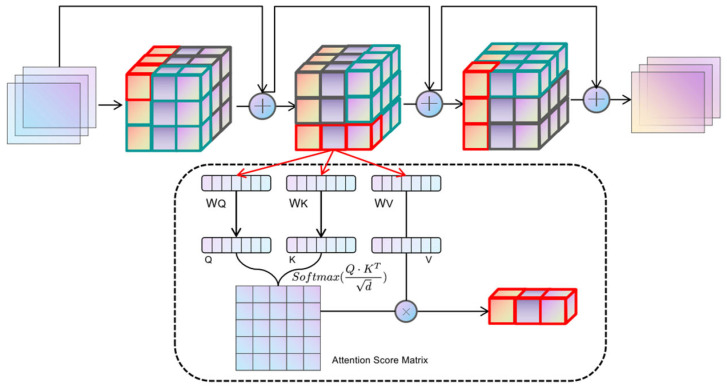
The schematic of IAF-Attention. The input image sequentially reshapes the tensor shape along different orthogonal directions and computes the attention.

Where silu denotes the Sigmoid linear cell activation function [39] and t denotes the number of iterations. The core idea is to decompose the 3D spatial attention into serialized local attention computations along three orthogonal directions (height H, width W, and depth D). Unlike window-based attention mechanisms (e.g., Swin Transformer), which approximate global interactions through shifted local windows, IAFT processes attention sequentially along each full axis. This sequential axial processing allows for the direct capture of long-range dependencies along individual anatomical axes. The iterative nature of IAFT (Equation (5)), where the output of one axial attention pass becomes the input for the next (after residual connection and SiLU), enables the integration of contextual information from all three dimensions. This iterative refinement across axes helps to preserve and model complex 3D geometric structures and inter-dependencies more effectively than factorizing attention independently without iteration or relying solely on local window interactions, particularly for elongated or complexly shaped tumors in 3D space. This approach significantly reduces computational complexity from $O(N^{2})$ for full 3D self-attention to $O(N^{4/3})$ for high-resolution 3D data, while enhancing the model’s capacity for geometric modeling of anatomical structures by maintaining spatial localization and capturing extensive contextual cues. The overall operation of Iterative Axial Factorization Attention is shown in Figure 4.

### 3.4. Multi-Scale Feature Fusion Decoder

In order to effectively integrate the multi-resolution features $\left(C_{1},C_{2},C_{3},C_{4}\right)$ of the encoder output, a lightweight hybrid upsampling decoder is designed in this study. The decoder accomplishes accurate pixel-level prediction through layer-level feature alignment and channel attention weighting with progressive upsampling.

For the four-stage feature output from the encoder, upsampling is performed using a combination of transposed convolution and trilinear interpolation to ensure that the spatial dimensions of each feature map are aligned to a uniform resolution. The upsampled features undergo a series of processing steps, beginning with joint channel-space optimization via a lightweight axial attention module. This module involves the spreading of features into a sequence form, their input into IAFT to compute cross-positional dependencies, the preservation of low-frequency anatomical structural information via residual concatenation, and the enhancement of nonlinear representativeness using the SILU activation function.

The aligned multi-scale features are spliced along the channel dimension and compressed to a uniform dimension by 1 × 1 convolution. A progressive upsampling strategy is finally adopted, which entails the restoration of resolution to the fused feature maps using transposed convolution. This is followed by the refinement and restoration of edge details to the original resolution using trilinear interpolation. Ultimately, the category probability maps are output through point-by-point convolution.

MSFFD employs a phased feature processing strategy to circumvent the memory bottleneck that arises from concurrent processing of full-resolution features. Axial attention is incorporated into the decoding process to preserve anatomical consistency and augment the modeling capability of the tumor boundary region. The computational volume of the decoder component is 3.71 GFLOPs, and the number of parameters is only 167,940, which accounts for a mere 3.32% of the total parameters of the model. This is indicative of the design concept of lightweight computing.

### 3.5. Loss Function

#### 3.5.1. Dice Loss

The Dice Loss function is among the most frequently employed loss functions in the domain of medical image segmentation, with its formula specified as follows:

(6) $$L_{\mathrm{Dice}}(y_{i},p_{i})=1-\frac{2\sum_{i}y_{i}p_{i}+\epsilon }{\sum_{i}\left(y_{i}+p_{i}\right)+\epsilon }$$

In this context, $y_{i}$ represents the true label, $p_{i}$ denotes the predicted probability, and i denotes the sample index. The Dice Loss has been demonstrated to be particularly effective in addressing the category imbalance problem. In the context of brain glioma segmentation, the tumor region is typically considerably smaller than the non-tumor region, leading to a pronounced category imbalance in the dataset. The Dice coefficient, a metric of similarity between two sets, is employed to quantify this imbalance. The Dice Loss function, which prioritizes the overlapping region over the precise classification, effectively mitigates the issue of significant imbalance between positive and negative samples in the glioma segmentation task.

#### 3.5.2. Cross Entropy Loss

The cross-entropy loss function is another widely used loss function for multi-category classification problems, with the formula definition shown in Equation (7):

(7) $$L_{\mathrm{CE}}(y_{i},p_{i})=-\frac{1}{N}\overset{N}{\underset{i=1}{\sum }}[y_{i}\ln p_{i}+(1-y_{i})\ln(1-p_{i})]$$

In this equation, $y_{i}$ denotes the true label, $p_{i}$ denotes the predicted probability, i denotes the sample index, and N denotes the total number of samples. The cross-entropy loss function is designed to promote accurate classification at the pixel level, a critical component for precise glioma localization.

#### 3.5.3. Training Policy

The cross-entropy loss function and the Dice Loss function are complementary in their approaches to the problem. The cross-entropy loss function emphasizes the differences in probability distributions, while the Dice Loss function concentrates on the size of the overlapping regions [40]. This complementarity allows for the combination of these elements, resulting in a more comprehensive enhancement of performance.

In the context of glioma segmentation tasks, it is often challenging for a single loss function to simultaneously satisfy multiple segmentation requirements. To harness the strengths of both the Dice Loss and the cross-entropy loss functions, a strategy of weighting the two loss functions is adopted. The joint loss function is defined as Equation (8):

(8) $$L_{\mathrm{Combined}}=\lambda_{1}L_{\mathrm{Dice}}+\lambda_{2}L_{\mathrm{CE}}$$

where $\lambda_{1}$ and $\lambda_{2}$ are weighting coefficients that balance the contributions of the two loss functions. By adjusting these weights, one can flexibly control the importance the model assigns to different loss terms.

### 3.6. Statistical Analysis

The performance of our proposed model and other compared methods was primarily evaluated using the Dice Similarity Coefficient (DSC) and the 95% Hausdorff Distance (HD95) for the enhanced tumor (ET), tumor core (TC), and whole tumor (WT) regions. For the quantitative results reported in our experiments (e.g., Section 4.2, Table 1 and Table 2; Section 4.3, Table 3), mean values and their corresponding 95% confidence intervals (CIs) were calculated to provide a measure of the precision of our estimates.

In the ablation study (Section 4.4, Table 4), paired-sample *t*-tests were employed to assess the statistical significance of differences in Dice scores for ET, TC, and WT regions between different model configurations (baseline model and models with incrementally added components). A *p*-value less than 0.05 was considered statistically significant (*), and a *p*-value less than 0.01 was considered highly statistically significant (**).

All statistical analyses, including the calculation of performance metrics and hypothesis testing, were performed using Python (version 3.11). Specifically, the NumPy library (version 1.26.4) was utilized for numerical operations and data handling, and the SciPy library (version 1.15.2, specifically the ‘scipy.stats.ttest_rel’ function) was used for conducting the paired-sample *t*-tests.

## 4. Results and Discussion

### 4.1. Experimental Setup

Our model operates on a platform equipped with a single NVIDIA RTX 3080 (10 GB VRAM), and the model code is implemented using the PyTorch (version 2.3.1) framework. We conduct experiments using two publicly available datasets: BraTS2020 [2] and FeTS2022 [41]. Each sample contains four modalities: T1, T2, T1ce, and Flair sequences. The BraTS2020 dataset includes MRI data from 369 patients diagnosed with various types of high-grade gliomas (HGGs) and low-grade gliomas (LGGs). The training set of FeTS2022 comprises a total of 1286 samples, which include MRI scans of brain tumors sourced from multiple centers. These scans are radiologically characterized as glioblastomas (GBMs); specifically, the FeTS dataset is a subset of GBM cases from the ongoing evaluation of BraTS. For both the BraTS2020 and FeTS2022 datasets, we partitioned the data into training, validation, and test sets in an 8:1:1 ratio. We selected the model exhibiting optimal performance based on validation set results and subsequently evaluated the final performance using the test set. During the training process, all scanned images were resized to a voxel dimension of 128 × 128 × 128, focusing on the region of interest. We applied data augmentation techniques to the input images in the training set, including random flipping (30% probability) and random rotation (50% probability, with an angle of ±30°) combined with noise. We set the batch size to two and the initial learning rate to 2 × 10^−4^, training the model for 400 epochs using the AdamW [42] optimizer. Additionally, we employed the ‘CosineAnnealingWarmRestarts’ learning rate strategy, which restarts the learning rate to the initial value at the beginning of each cycle (every 20 epochs) and subsequently decreases it to the minimum learning rate through cosine decay during the cycle, which we set to 1 × 10^−5^ in our experiments.

### 4.2. Comparison with State-of-the-Art Models

Our model was experimentally compared with a variety of techniques that encompass both classical models and recent state-of-the-art (SOTA) approaches, including 3D UNet [20], AttentionUNet [24], SegResNet [43], nnUNet [7], DiNTS [44], TransUNet [8], TransBTS [14], UNETR [9], nnFormer [23], DAUnet [45], Segformer3D [32], VcaNet [46], and CR-Swin2-VT [47]. All implementations were supplied by the authors, and all model setups and hyperparameters were maintained uniformly to guarantee an equitable comparison. In order to obtain the average value for all regions, the evaluation metrics compare the models’ Dice coefficients and 95% Hausdorff distance (HD95) over three distinct region types: enhanced tumor (ET), tumor core (TC), and whole tumor (WT). The quantitative comparison results of the various approaches on the BraTS2020 dataset are shown in Table 1, and the quantitative comparison findings on the FeTS2022 dataset are shown in Table 2.

**Figure 5:**
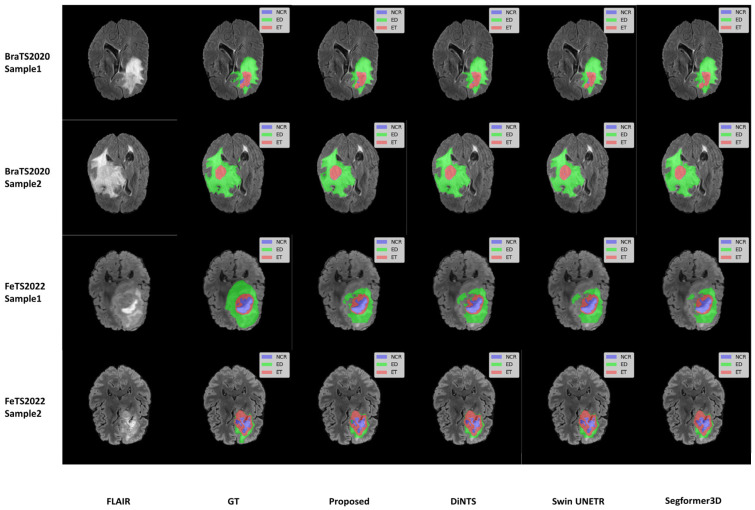
Qualitative visualization of brain tumor segmentation results on selected axial slices from the BraTS2020 and FeTS2022 datasets. For each sample, the FLAIR MRI sequence is shown first, followed by the Ground Truth (GT) segmentation and predictions from our proposed WIAF model, DiNTS, Swin UNETR, and Segformer3D. In the segmentation masks, tumor subregions are color-coded as follows: enhancing tumor (ET) in red, peritumoral edema (ED) in green, and necrotic and non-enhancing tumor core (NCR/NET) in blue.

We selected two samples from each dataset of BraTS2020 and FeTS2022 for qualitative analysis using the methods proposed in this paper, specifically DiNTS, Swin UNETR, and Segformer3D. The results of the visualization are presented in Figure 5. To facilitate presentation, the 3D medical images will be displayed using a grayscale map of intermediate 2D slices along the depth dimension, with different tumor subregions distinguished by varying colors. Each tumor part is represented by a corresponding color. We chose the Flair sequence image as the primary reference point, while the Ground Truth (GT) indicates the segmented region of the label mask.

**Figure 6:**
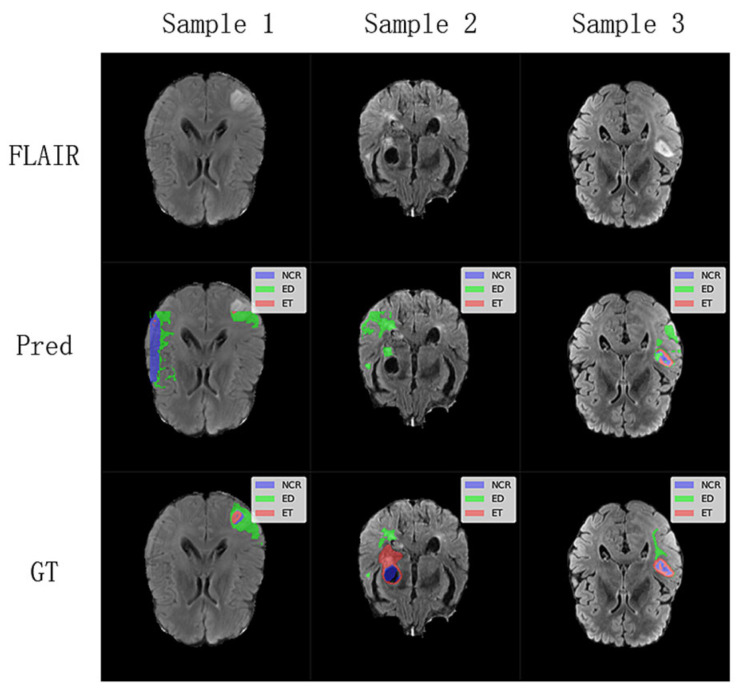
Analysis of challenging segmentation cases from the BraTS2020 and FeTS2022 testing dataset. The first row displays the FLAIR sequence (middle slice) of three distinct subjects where our model encountered difficulties. The Pred row shows the corresponding segmentation predictions from the WIAF model, and the GT row presents the ground truth labels.

To further understand the limitations of our proposed model, we investigated challenging cases where segmentation performance was suboptimal, as illustrated in Figure 6. These cases, identified through an analysis of outliers in our test set quantitative metrics, highlight specific scenarios where the model struggles. These challenging cases suggest that while our lightweight model performs well on average, its performance can be compromised in images with very low contrast between tumor and healthy tissue, or for particularly heterogeneous or small tumor sub-regions. The model’s efficiency, achieved through a reduced parameter count, might lead to a diminished capacity to learn highly complex and subtle feature variations present in these difficult instances. Future work could focus on targeted data augmentation for such low-contrast scenarios or a more sophisticated feature enhancement module to improve robustness in these specific failure modes.

Our proposed model achieves competitive performance on both datasets, We use *n* to represent the number of samples in the test set. Specifically, on the BraTS2020 test set (*n* = 38), for the ET, TC, and WT regions, our model obtained mean Dice scores of 76.8% (95% CI: 68.5–84.8%), 87.8% (95% CI: 85.4–90.6%), and 90.5% (95% CI: 88.8–92.3%), respectively. The corresponding mean HD95 values were 5.26 mm (95% CI: 1.65 mm–8.86 mm) for ET, 7.00 mm (95% CI: 3.60 mm–10.40 mm) for TC, and 7.03 mm (95% CI: 4.38 mm–9.69 mm) for WT.

On the larger FeTS2022 test set (*n* = 126), our model demonstrated mean Dice scores of 86.1% (95% CI: 82.2–89.9%) for ET, 92.0% (95% CI: 90.7–93.3%) for TC, and 93.4% (95% CI: 92.7–94.2%) for WT. The HD95 results were 2.12 mm (95% CI: 0.39 mm–3.85 mm) for ET, 3.72 mm (95% CI: 2.99 mm–4.44 mm) for TC, and 6.64 mm (95% CI: 4.60 mm–8.68 mm) for WT. These confidence intervals provide a measure of the precision of our mean estimates.

This advantage may arise from the adaptive wavelet decomposition, which preserves high-frequency details, enabling the model to delineate tumor boundaries more accurately. Additionally, the iterative axial attention mechanism mitigates the high computational cost associated with global self-attention by decomposing 3D spatial relations while retaining the capability to perceive a broad range of structures. In the ET region, our model’s Dice coefficient is comparatively lower than some heavier counterparts like DAUnet and Swin UNETR. This can be attributed to several factors inherent to lightweight designs and the nature of ET: these regions are often small, possess indistinct boundaries, and exhibit high heterogeneity. Our model’s lightweight architecture, while beneficial for computational efficiency, inherently involves a trade-off, potentially limiting the depth of feature extraction hierarchies required to capture extremely fine-grained details or subtle textural differences characteristic of small ET lesions. This is further corroborated by the Hausdorff distance for ET, which, being higher, suggests challenges in precise boundary localization for these smaller structures. However, the model’s strong performance in TC and WT regions underscores its efficacy for larger, more structurally defined tumor components. However, the model performs well in the TC and WT regions, reflecting its superiority in larger structures. Overall, the proposed method exhibits advantages in delineating tumor boundaries, particularly in regions with irregular tumor morphology. It consistently generates high-quality segmentation results across different slices and datasets, demonstrating strong performance consistency and robustness.

### 4.3. Quantitative Analysis of Lightweight Design

To validate the lightweight advantage of the proposed model, Table 3 compares the number of parameters, computational volume, inference speed, and average Dice coefficient across different methods using the BraTS2020 dataset. The proposed model requires only 5.23 million parameters with 9.75 GFLOPs of computation and achieves an average inference time of 289.47 ms per 3D patient volume on a single RTX 3080 GPU. This represents an 89.6% reduction in parameter count and a 99.2% reduction in computational load compared to DiNTS, while maintaining state-of-the-art segmentation performance. Notably, although Segformer3D has fewer parameters, it yields lower average Dice coefficients than the proposed method, demonstrating that our model achieves a superior balance between accuracy and efficiency in lightweight design.

**Figure 7:**
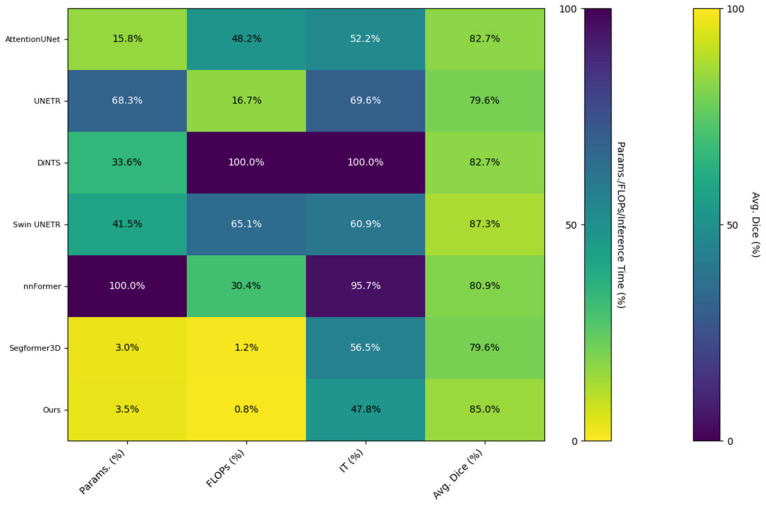
Matrix heatmap of the quantitative metrics from Table 3, comparing the computational efficiency and segmentation performance of various methods.

Figure 7 presents the matrix heat map derived from the results displayed in Table 3. We normalized the non-uniform scale metrics (Parameters, FLOPs, Inference Time) from Table 3, converting all metrics into percentile values ranging from 0 to 1 based on the maximum value for each metric. This transformation visualizes the lightweight design and actual performance of the model through matrix heatmaps: lighter colors indicate higher performance, while darker colors signify lower performance. The three indicators—Parameters, FLOPs, and Inference Time—are arranged in reverse order, meaning that a lower percentage indicates better performance. Conversely, the Dice indicator is plotted in a positive order, where a higher percentage reflects superior model performance, represented by brighter colors in the matrix heatmap. The visualization results in Figure 6 illustrate that our method effectively mitigates the challenges associated with model parameter count and computational redundancy in medical image segmentation tasks, achieving a high-quality balance between performance and efficiency.

### 4.4. Ablation Study

To verify the effectiveness of the method proposed in this paper, we conducted ablation experiments on the BraTS2020 dataset. Specifically, we removed the AWD-FD module from the proposed model and replaced it with 3D convolution for the downsampling operation. Additionally, we substituted the IAFT with a Transformer Block that implements standard multi-head self-attention. To address the issue of excessive 3D image size in the initial stage of the encoder, which can lead to memory overflow, we employed 3D convolution with a stride of two to downsample the image at this stage. This downsampling allows us to obtain the self-attention Key and Value components after size reduction. Subsequently, we calculated the attention using the original Query component to mitigate the problem of video memory overflow under high token length. Finally, in the decoder layer, we adopted transposed convolution for upsampling to restore the feature maps at different stages to a unified size, followed by feature fusion and output classification operations. The model resulting from all these modifications served as baseline B for our ablation experiment, to which we added various modules designed by us to validate the effectiveness of the proposed method.

All comparison results are derived from the test subset of BraTS2020. Table 4 illustrates the changes in DSC, parameter count, and computational load from the infrastructure to the complete model.

After adopting the standard 3D convolution downsampling, the average Dice coefficient decreased to 81.4%, with particularly poor performance observed in the TC region. This suggests that traditional downsampling approaches may lead to the loss of high-frequency details. The introduction of the MSFFD resulted in a significant reduction in both the number of parameters and computational load. However, the Dice coefficients across all regions experienced a decline; this indicates that an upsampling strategy alone is insufficient to fully capture cross-level feature correlations. In contrast, the addition of iterative axial decomposition attention led to a notable increase in the Dice coefficient for the TC region, which rose to 87.0%, while the WT region achieved a coefficient of 90.9%. This finding validates the effectiveness of axial attention in modeling long-range dependencies.

**Figure 8:**
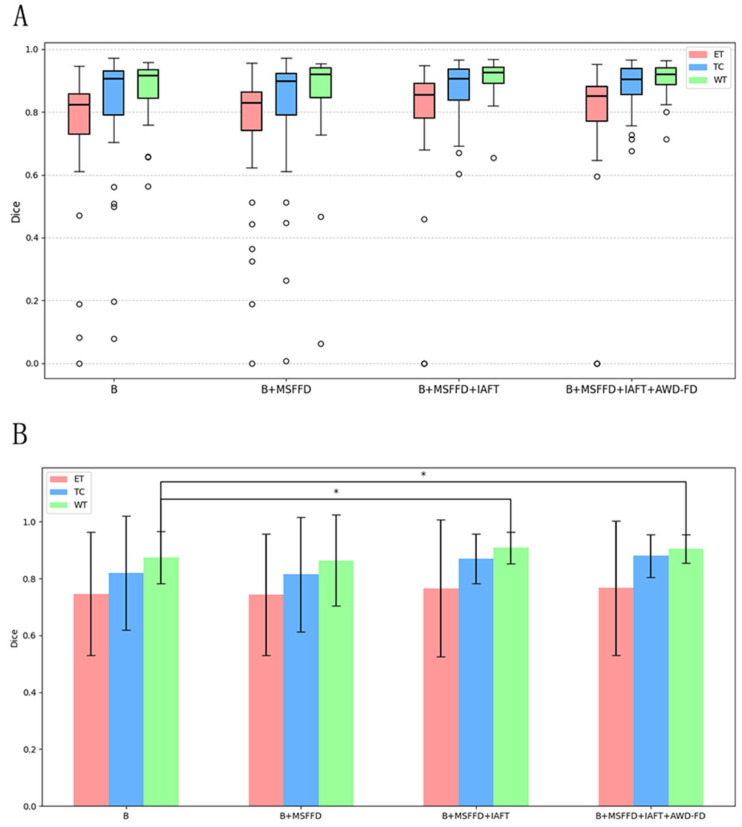
Ablation study results on the BraTS2020 test set. (**A**) Boxplots showing the distribution of Dice scores for ET, TC, and WT for the baseline model and with the progressive addition of modules. Circles represent outliers. (**B**) Bar chart depicting mean Dice scores with error bars for ET, TC, and WT across the ablation study configurations. Asterisks (*) indicate statistically significant differences (*p* < 0.05), determined by paired *t*-tests.

Figure 8 presents a detailed analysis of the ablation experiments, illustrating the incremental impact of the MSFFD, IAFT, and AWD-FD modules on segmentation performance using the BraTS2020 test set. The final integration of the AWD-FD module led to a further increase in the Dice coefficient for the TC region from 87.0% to 87.8% and the average Dice to 85.0% (Table 4). The impact of AWD-FD on preserving fine structural details is visually exemplified in Figure 8, where the model with AWD-FD demonstrates improved delineation of tumor core boundaries and internal heterogeneity compared to the model without it. This highlights the AWD-FD module’s contribution to better feature representation by preserving high-frequency information. As illustrated in Figure 8, the complete model also exhibits a more concentrated distribution of scores with fewer outliers.

**Figure 9:**
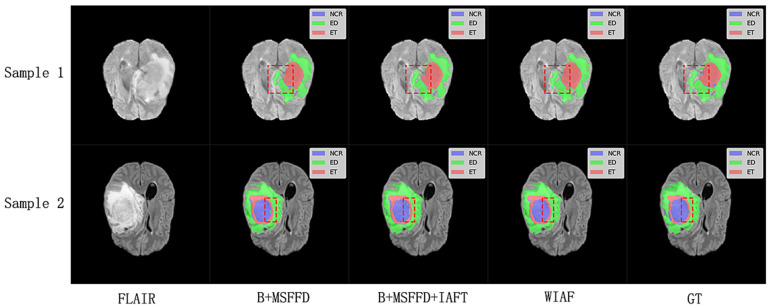
Qualitative comparison of ablation study results on a representative axial slice from the BraTS2020 testing dataset. The red dashed boxes highlight the key differences between the model predictions at different stages and the ground truth labels.

The visual results shown in Figure 9 further validate the quantitative findings of the ablation study. The model without IAFT and AWD-FD exhibits significant undersegmentation in the ED region compared to the ground truth labels, and the boundaries of the WT region are not defined with sufficient precision. After integrating the IAFT module, a significant improvement in tumor boundary segmentation was observed, highlighting the effectiveness of IAFT in modeling long-range dependencies and optimizing feature representations. Finally, the complete proposed model incorporating the AWD-FD module is the closest to the ground truth (GT). The AWD-FD module appears to further optimize segmentation by preserving finer details and improving contour accuracy, particularly in the ED region and at the interfaces between different tumor subregions. For example, the highlighted regions in Sample 2 indicate that the model successfully captured subtle infiltration patterns and reduced false positives within the tumor compared to the intermediate ablation stage. This qualitative evidence strongly supports the synergistic contribution of each proposed module toward achieving robust and accurate brain tumor segmentation.

## 5. Conclusions

This study proposes an innovative lightweight deep learning model for the efficient segmentation of brain tumors from 3D MRI scans. By introducing adaptive discrete wavelet decomposition and an iterative axial attention mechanism, our model significantly reduces computational complexity while maintaining high-precision segmentation capabilities. The experimental results demonstrate that this model achieves an average Dice coefficient of 85.0% and 88.1% for BraTS2020 and FeTS2022, respectively, reaching an advanced level compared to the state-of-the-art models. Additionally, it maintains a low parameter count and computational load, outperforming many existing complex models. This indicates that our method strikes a balance between performance and efficiency, possesses significant advantages in resource-constrained scenarios, and offers practicality for clinical practice.

However, this study has certain limitations. The current framework primarily focuses on Dice Loss and cross-entropy loss, which, while effective for overall segmentation accuracy, may not fully address the under-segmentation of small targets like ET regions due to class imbalance. For instance, focal loss, by down-weighting the loss assigned to well-classified examples, can encourage the model to focus more on hard-to-segment samples, which often include the small and indistinctly bounded ET regions. This could potentially alleviate the under-segmentation issue observed for ET, which is often attributed to class imbalance and its challenging characteristics. Future work will explore integrating focal loss variants or sensitivity-specific metrics (e.g., recall for ET) to further improve performance on these critical sub-regions.

Looking ahead, we intend to broaden the application of our model to other 3D medical imaging tasks to assess its generalization potential. Concurrently, investigating more flexible wavelet transform strategies or integrating multimodal data may yield additional performance enhancements. Additionally, optimizing the segmentation of small regions will emerge as a crucial research direction. This study, by integrating the frequency domain with the attention mechanism, has demonstrated the feasibility of a lightweight model for high-precision medical segmentation, thereby providing a theoretical foundation and direction for the future development of efficient and universal clinical tools.

## Figures and Tables

**Table 1:** Quantitative analysis of the BraTS2020 dataset. Dice coefficients and Hausdorff distances of ET, TC, and WT were used as evaluation metrics. The up arrow (↑) indicates that higher values are better, while the down arrow (↓) indicates that lower values are better.

Methods	Dice (%)↑				Hausdorff 95% (mm)↓			
	ET	TC	WT	Avg.	ET	TC	WT	Avg.
3D UNet	73.1	79.7	86.4	79.7	17.6	11.7	10.1	13.1
AttentionUNet	75.5	84.6	87.9	82.7	25.8	8.1	9.8	14.6
SegResNet	78.8	84.8	89.5	84.4	15.7	8.9	9.2	11.3
DiNTS	76.4	84.2	87.6	82.7	18.9	9.8	9.4	12.7
UNETR	74.0	79.8	85.0	79.6	27.8	10.5	9.9	16.1
TransBTS	78.7	81.7	90.9	83.8	17.9	9.8	5.0	10.9
Swin UNETR	82.9	88.6	90.3	87.3	26.1	16.7	8.0	16.9
nnFormer	71.5	79.6	91.8	80.9	16.9	8.8	7.0	10.9
DAUnet	78.6	83.0	89.8	83.8	14.4	6.6	6.7	9.2
Segformer3D	71.5	81.9	85.3	79.6	25.2	5.0	8.0	12.8
VcaNet	78.7	83.4	90.6	84.2	15.8	10.1	10.6	12.2
WIAF	76.8	87.8	90.5	85.0	27.1	6.6	6.3	13.3

**Table 2:** Quantitative analysis of the FeTS2022 dataset. Dice coefficients and Hausdorff distances of ET, TC, and WT were used as evaluation metrics. The up arrow (↑) indicates that higher values are better, while the down arrow (↓) indicates that lower values are better.

Methods	Dice (%)↑				Hausdorff 95% (mm)↓			
	ET	TC	WT	Avg.	ET	TC	WT	Avg.
nnUNet	83.7	87.5	92.6	87.9	22.4	10.6	3.6	12.2
UNETR	82.3	86.4	91.7	86.8	23.0	11.5	9.1	14.5
DiNTS	83.4	89.6	93.2	88.7	18.5	8.2	6.2	11.0
Swin UNETR	83.8	88.7	92.6	88.4	20.2	10.0	7.6	12.6
Segformer3D	78.7	88.2	91.5	86.1	19.5	6.6	6.8	11.0
CR-Swin2-VT	81.7	85.4	91.4	86.2	14.8	11.2	3.9	10.0
WIAF	82.7	89.1	92.5	88.1	18.6	8.0	5.9	10.8

**Table 3:** Quantitative analysis of lightweight design on the BraTS2020 dataset. This table compares parameter count (Params.), floating-point operations (FLOPs), average inference time per 3D patient volume (Inference Time), and average Dice coefficient (Avg. Dice). Inference time was measured on a single NVIDIA RTX 3080 GPU.

Method	Params. (M)	FLOPs (G)	Inference Time (ms)	Avg. Dice (%)
AttentionUNet	23.63	587.61	315.79	82.7
UNETR	102.06	203.32	421.05	79.6
DiNTS	50.22	1219.52	605.26	82.7
Swin UNETR	62.00	793.92	368.42	**87.3**
nnFormer	149.34	370.43	578.95	80.9
Segformer3D	**4.53**	14.53	342.11	79.6
Ours	5.23	**9.75**	**289.47**	85.0

**Table 4:** Ablation study of the proposed model on the testing dataset of BraTS2020. The Dice score on ET, TC, and WT were evaluated to demonstrate the contribution of each component.

Method	Params.(M)	FLOPs (G)	Dice (%)↑			
			ET	TC	WT	Avg.
B	8.67	290.35	74.7	82.0	87.4	81.4
B + MSFFD	4.23	7.52	74.4	81.5	86.4	80.8
B + MSFFD + IAFT	5.19	8.50	76.6	87.0	**90.9**	84.8
B + MSFFD + IAFT + AWD-FD	5.23	9.75	**76.8**	**87.8**	90.5	**85.0**

## Data Availability

The original data presented in the study are openly available in the Multimodal Brain Tumor Segmentation Challenge 2020 repository at https://www.med.upenn.edu/cbica/brats2020/data.html, accessed on 3 June 2025 and Federated Tumor Segmentation (FeTS) 2024 Challenge repository at https://www.synapse.org/fets2024, accessed on 3 June 2025.

## Supplementary Materials

The full implementation of the proposed framework is publicly available at https://github.com/ZyySagit/WIAF-Brain-Tumor-Segmentation, accessed on 3 June 2025.
